# Localization Algorithm with On-line Path Loss Estimation and Node Selection

**DOI:** 10.3390/s110706905

**Published:** 2011-07-01

**Authors:** Albert Bel, José López Vicario, Gonzalo Seco-Granados

**Affiliations:** Signal Processing for Communications and Navigation Group, Telecommunications and Systems Engineering Department, Universitat Autònoma de Barcelona, Edifici Q Campus de la UAB, Bellaterra (Cerdanyola del Vallès), Barcelona 08193, Spain

**Keywords:** wireless sensor networks, localization, cooperative, distributed, Received Signal Strength (RSS)

## Abstract

RSS-based localization is considered a low-complexity algorithm with respect to other range techniques such as TOA or AOA. The accuracy of RSS methods depends on the suitability of the propagation models used for the actual propagation conditions. In indoor environments, in particular, it is very difficult to obtain a good propagation model. For that reason, we present a cooperative localization algorithm that dynamically estimates the path loss exponent by using RSS measurements. Since the energy consumption is a key point in sensor networks, we propose a node selection mechanism to limit the number of neighbours of a given node that are used for positioning purposes. Moreover, the selection mechanism is also useful to discard bad links that could negatively affect the performance accuracy. As a result, we derive a practical solution tailored to the strict requirements of sensor networks in terms of complexity, size and cost. We present results based on both computer simulations and real experiments with the Crossbow MICA2 motes showing that the proposed scheme offers a good trade-off in terms of position accuracy and energy efficiency.

## 1. Introduction

In recent years, location estimation in wireless sensor networks (WSN) has raised a lot of interest from researchers [1–3]. In order to give sense to the measured data by a WSN, it is necessary in the majority of the environments to give the location of the nodes. The WSN localization techniques are used to obtain estimates of nodes position with initially unknown positions. In order to do that, those non-located nodes take the advantage of the knowledge of the positions of some sensors with known location (anchor nodes) and inter-node measurements. Those anchor nodes can obtain their locations by means of using a global positioning system (GPS), or by setting those nodes at known positions. Localization methods are normally divided in two phases [4]. The first phase consists in the estimation of distances between nodes. At the second phase, the localization algorithm computes the position of each node through different methods. Next, we present these two phases in detail:

### 1.1. Measurement Phase

In this subsection we present three different signal metrics used to obtained distances estimates.

- **Time Measurements** Distance estimates obtained through time measurements are usually estimated using different methods. It is possible to obtain the distance by means of using the time of arrival (TOA) of a signal [5]. When a node receives a message it extracts the distance through the measurement of the transmission time. Although a high accuracy can be achieved with this method, it is necessary to have a synchronized network with the same reference clock in all the nodes.
  Another method based on time measurements is the Time Difference of Arrival (TDOA). In [3], two different methods are presented in accordance with the nature of the time estimates. First TDOA method [6] is based in the measurement of the difference between the arrival time of the same signal at two receivers. This method assumes that the receiver locations are known and the two receivers are perfectly synchronized. The second TDOA method [7] eliminates the necessity of having a synchronized network. It uses a combination of two kind of signals, e.g., RF and ultrasonic signals. The time difference between the first and the second signal is used as an estimate of the one-way acoustic propagation time. In this case nodes require extra hardware in order to be able to transmit different signals.
  Finally, in order to avoid the necessity of a synchronized network or the use of different transmit signals, one can find in literature the Roundtrip Propagation time measurements [8]. In this method, one sender sends one signal to a node and this node retransmits the same signal to the sender node. Then this method measures the difference between the time when a signal is sent and the time when this signal comes back to the sender node. This time measurement is done with the same local clock, so it avoided the need of a synchronized network. Also, it is not necessary to send different signals. The major downside of this method is the necessity of a double transmission in order to obtain a time measurement and of knowing the time delay of transmission at the receiver node.
- **Angle Measurements** Methods using angle measurements are known as Angle or Direction of Arrival, AOA and DOA [9]. The angle is estimated with the use of directive antennas or array of antennas. The necessity of extra hardware is the major disadvantage of this method that could probably increase the size and cost of the nodes.
- **Received Signal Strength Measurements** In this case, the distance between two nodes is obtained by using the power of the received signal [10]. RSS-based distance estimations, in particular, are based on the well-known radio propagation path loss model. Compared to AOA or TOA-based techniques, this technique has become the most inexpensive because RSS signals can be obtained during normal transmissions. However, distance estimates obtained through RSS measurements present less accuracy than the other two methods.

### 1.2. Location-Update Phase

The second phase uses the previous distance estimates to start the location procedure. Existing algorithms could be classified as centralized versus distributed, and non-cooperative versus cooperative. In centralized algorithms, a central processor receives all the information and calculates the position of all the network nodes. The central processor probably will not have any processing limitation, but on the other hand, this solution limits the scalability of the system. Hence, centralized solutions are not very attractive for large scale networks, where distributed algorithms are preferred. The second classification differentiates between the cooperative and non-cooperative techniques [11]. We assume that a network is formed by nodes with known position, called reference or anchor nodes, and nodes without knowing their position, called non-located nodes. In non-cooperative techniques, non-located nodes are only able to communicate with reference nodes. On the other hand, cooperative techniques allow a non-located node to communicate with any node either anchor or non-located. In general, cooperative techniques can increase localization performance in terms of both accuracy and coverage.

### 1.3. Contribution

In this paper, we focus on a cooperative distributed localization method based on RSS measurements. RSS measurements become the simplest choice in order to reduce the complexity and the cost of the nodes. Although distance estimates obtained through RSS measurements have a lower accuracy, we try to reduce the adverse effects introduced by modelling problems. The choice of a distributed strategy is motivated by the desire to reduce the necessity of transmitting all the network information to a central node. With this adoption each node modifies in the second phase its own state through those estimated metrics and the nodes state information. We also adopt a cooperative technique. Although cooperative techniques could increase localization accuracy, cooperation with distant nodes could introduce a higher degradation in the estimate depending on the estimation method used [12]. This is because the error introduced in the measurements can be multiplicative to the distance when RSS measurements are considered [13]. In addition, allowing the cooperation with more nodes increase the consumption of energy. The introduction of node selection strategies allow to the localization algorithms to minimize the energy consumption and the cooperation with further nodes while maintaining location accuracy.

Other sources of error that affect RSS-based distance estimations are shadowing and multipath signals, which complicate the modelling of the channel that nodes need to know a *priori*. A previous measurement campaign is usually carried out in order to obtain a proper model. The introduction of an on-line estimation of the propagation model helps to the RSS-based method to adapt to the scenario without the necessity of an off-line calibration. We propose a cooperative distributed positioning algorithm that dynamically estimates the propagation model that best fits the propagation environment by means of RSS measurements only.

Furthermore, two node selection criteria are proposed in order to reduce the number of cooperative nodes. By doing so, the scheme only selects those nodes providing accurate distance measurements. Another benefit is that the energy consumption is reduced. As a result, accuracy *vs.* energy consumption trade-off is significantly improved. As shown in the paper, experimental results carried out with Crossbow MICA2 motes validates the proposed scheme.

## 2. RSS-Based Distance Estimation

**Figure 1.**
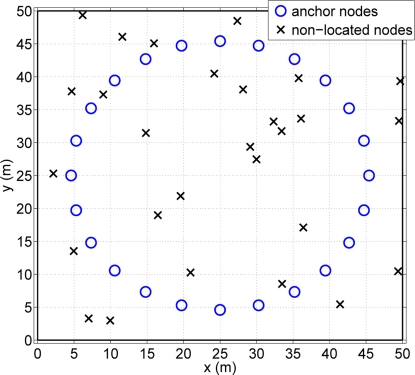
Example of an scenario deployment.

Let us consider a wireless sensor network with *N* nodes. There are *N*_1_ nodes, whose exact locations are known (anchor nodes). The rest of the nodes *N*_2_ = *N* – *N*_1_ do not know their position (non-located nodes). The main goal is to estimate the location of the non-located nodes with the help of anchor nodes and the rest of nodes in the network by means of a cooperative strategy. Concerning the anchor nodes placement, we follow the approach presented in [14], where it is shown that the best anchor placement is a centered circumference with radius equal to the root-mean-square (rms) of the non-located nodes distances to the center. One can see in Figure 1 an example of the scenario deployment.

As previously commented, we consider an RSS-based distributed cooperative algorithm for location estimation. The first phase of the algorithm consists in obtaining internode distances through RSS measurements. Power received is modelled through the well-known radio-propagation path loss and shadowing model [12]. Received Signal Strength (RSS) can be expressed as the power received in node j from a signal transmitted by node *i*, *P_ij_*, as:

(1) $${\mathrm{RSS}}_{\mathrm{ij}}=P_{\mathrm{ij}}=P_{0}-10\alpha_{\mathrm{ij}}{\text{log}}_{10}d_{\mathrm{ij}}-v_{\mathrm{ij}}\;(\mathrm{dBm})$$

where *P*_0_ is the power received in dBm at 1 m distance, *d_ij_* is the distance between nodes *i* and *j*, parameter *α_ij_* is the path loss exponent and *v_ij_* ∼ 𝒩 (0, $\sigma_{v}^{2}$) represents log-normal shadow-fading effects, where the value of the standard deviation *σ_v_* depends on the characteristics of the environment. Given the power received *RSS_ij_* in Equation (1), an ML estimate of the actual distance can be derived as:

(2) $$\delta_{\mathrm{ij}}=10^{\frac{P_{0}-{\mathrm{RSS}}_{\mathrm{ij}}}{10\alpha_{\mathrm{ij}}}}$$

## 3. On-Line Localization Algorithm

Once the relative distances between nodes are obtained, the position estimates for each non-located node are estimated by means of the least squares criterion. Position estimates are calculated by obtaining the set of non-located node positions and path loss exponents that minimize the difference between estimated distances at the first phase and the distances computed using such position estimates. In particular, the problem consists in minimizing the following cost function:

(3) $$C_{\mathrm{LS}}(\text{x},\text{α})={\sum_{i=1}^{N_{2}}\sum_{j\in S_{i}}\left(\delta_{\mathrm{ij}}\left(\alpha_{\mathrm{ij}}\right)-d_{\mathrm{ij}}\left({\text{x}}_{i},x_{j}\right)\right)}^{2}$$

where *d_ij_*(**x***_i_*, **x***_j_*)=||**x***_i_* − **x***_j_*|| is the distance between nodes *i* and *j*, calculated with the estimated position (or real coordinates if node *j* is an anchor) of nodes *i* and *j*, *S_i_* is the group of nodes (anchor and non-located) that cooperates in the position estimation of non-located node *i*, **x** are the coordinates of nodes, and ***α*** the set of all path loss exponents of all links. The minimization of the cost function of Equation (3) is carried out in a distributed fashion. We adopt a distributed strategy due to its scalability and robustness. We obtain the minimization of Equation (3) by computing its derivative with respect to *x_i_*:

(4) $$\frac{\partial C_{\mathrm{LS}}}{\partial {\text{x}}_{i}}=\sum_{j\in S_{i}}\frac{\partial {\left(\delta_{\mathrm{ij}}-d_{\mathrm{ij}}\right)}^{2}}{\partial {\text{x}}_{i}}+\sum_{k\in S_{i}}\frac{\partial {\left(\delta_{\mathrm{ij}}-d_{\mathrm{ki}}\right)}^{2}}{\partial {\text{x}}_{i}}$$

Notice that the second part of the sum can be omitted. This is because reciprocal channels are not assumed and, then, the value of *δ_ki_* cannot be estimated at node *i*. As a result, the cost function adopted by each node can be rewritten as:

(5) $$C_{\mathrm{DLS}}\left({\text{x}}_{i},\alpha_{i1},\alpha_{i2},\ldots ,\alpha_{i}N_{S_{i}}\right)=\sum_{j\in S_{i}}{\left(\delta_{\mathrm{ij}}\left(\alpha_{\mathrm{ij}}\right)-d_{\mathrm{ij}}\left({\text{x}}_{i},{\text{x}}_{i}\right)\right)}^{2}$$

Here, the objective is to minimize the difference between both distances by optimizing the node coordinates and the set of path loss exponents. The node coordinates (**x_i_**) and the set of all path loss exponents (*α_ij_* ∀ *j ɛ S_i_*) affect the computation of both distances, *d_ij_* and *δ_ij_*, respectively.

In order to solve the cost function of Equation (5) we adopt the Gauss-Seidel algorithm [15]. This non-linear algorithm is based on a circular iterative optimization with respect to one set of variables while maintaining the rest of the variables fixed. Hence, the minimizations are carried out successively for each component. If we consider a generic cost function *F* that depends on a set of variables *β*, the desired minimization of *F* is formally defined as [15]:

(6) $$\beta_{i}\left(t+1\right)=\text{arg}\;\underset{\beta_{i}}{\text{min}}F\left(\beta_{1}\left(t+1\right),\ldots ,\beta_{i-1}\left(t+1\right),\beta_{i},\beta_{i+1}\left(t\right),\ldots ,\beta_{m}\left(t\right)\right)$$

At time instant *t* + 1, the value of the component *β_i_* is optimized. Components from *β*_1_ to *β_i_*_−1_ have been already minimized whereas components from *β_i_*_+1_ to *β_m_* (being *m* the total number of components) have not been optimized. In our problem, cost function Equation (5) depends on the components (**x***_i_*, ***α****_S_i__*). By using the Gauss-Seidel approach, we can divide the optimization in two steps: firstly a minimization of the cost function by means of optimizing the node coordinates (fixing the path loss exponents) is carried out; secondly, another minimization is done by means of the optimization of the path loss exponent (fixing the nodes coordinates). As the convergence of the non-linear Gauss-Seidel algorithm can be established using a descent approach (see [15]), both minimizations are carried out through a gradient descent mechanism. Furthermore, the gradient descent method is a low computational method and it is distributable and scalable. It is a method that does not necessarily deliver the global optimal solution in non-convex problems, as it is the case of the problem presented here, unless a good starting point is available. For that reason, a study of the initial point’s impact on the behaviour of the algorithm is presented later on. Our results show that in practice, with an appropriate initialization, the gradient descent technique converges to a good solution.

### 3.1. Optimization of the Node Coordinates

We initially obtain a minimization of the cost function by optimizing the nodes coordinates. We first maintain all the *α_ij_* fixed ∀ *j ∈ S_i_*. As *d_ij_*(**x***_i_*, **x***_j_*) = *f*(**x_i_**, **x_j_**)=||**x_i_** − **x_j_**|| depends on the coordinates **x***_i_* the gradient of the cost function is:

(7) $$\begin{array}{r} \nabla_{{\text{x}}_{\text{i}}}C_{\mathrm{DLS}}\left({\text{x}}_{i},{\text{α}}_{S_{i}}\right)=\nabla_{{\text{x}}_{\text{i}}}\left(\sum_{j\in S_{i}}{\left(\delta_{\mathrm{ij}}\left(\alpha_{\mathrm{ij}}\right)-\left\|{\text{x}}_{i}-{\text{x}}_{i}\right\|\right)}^{2}\right)\\ =\sum_{j\in S_{i}}\left(\delta_{\mathrm{ij}}\left(\alpha_{\mathrm{ij}}\right)-d_{\mathrm{ij}}\left({\text{x}}_{i},{\text{x}}_{j}\right)\right){\text{e}}_{\mathrm{ij}} \end{array}$$

where ${\text{e}}_{\mathrm{ij}}=\frac{{\text{x}}_{i}-{\text{x}}_{j}}{\left\|{\text{x}}_{i}-{\text{x}}_{j}\right\|}$ is the unit vector that takes the orientation between the node *i* and node *j*. So, the estimate of **x***_i_*, can be iteratively computed by using the gradient descent algorithm as follows:

(8) $${\hat{\text{x}}}_{\text{i}}\left(t+1\right)={\hat{\text{x}}}_{\text{i}}\left(t\right)+\gamma \sum_{j\in S_{i}}\left(\delta \left(\alpha_{\mathrm{ij}}\right)-d\left({\text{x}}_{i},{\text{x}}_{j}\right)\right){\text{e}}_{\mathrm{ij}}$$

The use of a gradient descent method requires initial values for the position estimates (**◯**(*t* = 0)). Several methods can be found in the literature to obtain appropriate initial values. In that direction, MultiDimensional Scaling (MDS) is proposed in [12] to obtain such values. It is a good option but it increases the computational cost. Another option can be based on a random initialization. It is a simple method but a higher number of iterations is required for algorithm convergence. We propose to initialize each **◯***_i_*(0)as the weighted mean of the coordinates of the *n_anch_* nearest anchor nodes. When the node *i* sets its own group *S_i_* (see Section 4), it has to find the nearest anchor nodes and computes a weighted mean as:

(9) $${\hat{\text{x}}}_{i}\left(t=0\right)=\sum_{a=1}^{n_{\mathrm{anch}}}x_{a}\left|\frac{{\mathrm{RSS}}_{\mathrm{ia}}}{\sum_{a=1}^{n_{\mathrm{anch}}}{\mathrm{RSS}}_{\mathrm{ia}}}\right|$$

where **x***_a_* are the coordinates of the anchor *a* ∈ *S_i_*.

**Figure 2.**
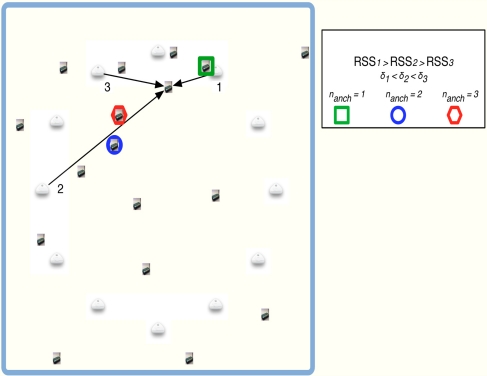
Example of an initial position with different values of *n_anch_*.

**Figure 3.**
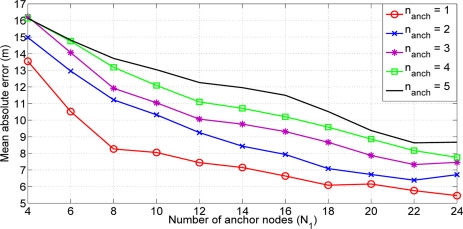
Mean absolute error *vs.* number of anchor nodes (*N*_1_).

With this estimation we could achieve an initial value closer to the final solution without an important increase of the computational cost. As we are assuming an equal path loss, the selection of the nearest anchor could not reflect the reality (see Figure 2). For that reason, we present an study of the influence of the number of anchor nodes used in the initial estimation of the non-located nodes inside the network. Figure 3 shows the results obtained. One could observe that better results are obtained when a number of anchor nodes equal to 1 is used to calculate the weighted mean. We use a fixed number of *n_anch_* =1 in the sequel. At first sight, this result could seem strange because, normally, it is better to use as many nodes as possible. But at the initial time instant of our algorithm we have not still estimated the path loss exponent values. Hence, the higher *RSS_ij_*, the lower distance estimate *δ_ij_*. If the number of anchors nodes *n_anch_* has a greater value, we can increase the probability of having a further anchor node. Hence, we can estimate an initial position far away from the real position. For that reason it is better to select only the anchor node with the highest RSS.

### 3.2. Optimization of the Path Loss Exponents

As previously commented, the objective of this paper is the improvement of RSS-based localization algorithm. However, the use of RSS measurements requires an accurate scenario modelling. In order to obtain a good model, a thorough previous measurement campaign should be carried out. A change in the environment becomes in the necessity of repeating the measurement campaign. By adopting an on-line estimation of the path loss exponent, the algorithm can be adapted to the scenario without the need of performing another measurement campaign. With on-line estimation we can also avoid the equal path loss exponent assumption for all links.

Following the Gauss-Seidel approach, we now minimize the cost function of Equation (5) fixing the nodes coordinates **x**. As the $\delta_{\mathrm{ij}}=f\left(\alpha_{\mathrm{ij}}\right)=10^{\frac{P_{0}-RSS_{\mathrm{ij}}}{10\alpha_{\mathrm{ij}}}}$ depends on the path loss exponent *α_ij_*, the cost function becomes:

(10) $$C_{\mathrm{DLS}}\left({\text{x}}_{i},\alpha_{S_{i}}\right)={\sum_{j\in N_{S_{i}}}\left(10^{\frac{P_{0}-{\mathrm{RSS}}_{\mathrm{ij}}}{10\alpha_{\mathrm{ij}}}}-d_{\mathrm{ij}}\left({\text{x}}_{i},{\text{x}}_{j}\right)\right)}^{2}$$

As each *δ_ij_* is a function of *α_ij_*, but not on *α_ik_* with *k* ≠ *j* ∈ *S_i_* (independent links between nodes), we can minimize cost function for each individual link. In that case, the fixed variables are the coordinate estimates and the rest of the path loss exponents (*α_ik_* ∀ *k* ≠ *j*). The gradient of cost function of Equation (10) is:

(11) $$\begin{array}{lll} \nabla_{\alpha_{\mathrm{ij}}}C_{\mathrm{DLS}}({\text{x}}_{i},{\text{α}}_{S_{i}}) & = & \nabla_{\alpha_{\mathrm{ij}}}({\left(10^{\frac{P_{0}-{\mathrm{RSS}}_{\mathrm{ij}}}{10\alpha_{\mathrm{ij}}}}-d_{\mathrm{ij}}({\text{x}}_{i},{\text{x}}_{j})\right)}^{2})\\ & & =-\;\text{log}(10)\frac{P_{0}-{\mathrm{RSS}}_{\mathrm{ij}}}{5}\frac{1}{\alpha_{\mathrm{ij}}^{2}}\delta_{\mathrm{ij}}(\delta_{\mathrm{ij}}-d_{\mathrm{ij}}({\text{x}}_{\text{i}},{\text{x}}_{\text{j}})) \end{array}$$

Each node estimates their own path loss exponents for all the links. This is the major difference between our proposal and the proposed method in [16], which is centralized. Our proposal is a distributed method that minimizes the cost function through an iterative gradient descent strategy. Finally, it is worth noting that restrictions are applied to the set of solutions for *α_ij_*. More specifically, the following feasible set of solutions is considered in order to avoid undesirable results:

(12) $$\alpha_{\min}\leq \alpha_{\mathrm{ij}}\leq \alpha_{\max}$$

where we have considered that *α_min_* and *α_max_* are equal to 2 and 5, respectively. This selection is based on the knowledge of the kind of deployment scenario.

### 3.3. Localization Algorithm

With the obtained path loss exponent estimations, we can update distance estimates *δ_ij_* and recalculate coordinates estimates **◯***_i_*. Following the Gauss-Seidel approach, both previous procedures are repeated in a circular fashion until convergence. A scheme of the global procedure of the location algorithm can be found in the Algorithm 1, with *t_iter_*_1_ and *t_iter_*_2_ being the required number of iterations to converge to the final solution.

**Algorithm 1:** LS Localization Algorithm with On-Line Path Loss Estimation

**for***t* = 1 to *t_iter_*_1_**do**
**Coordinate Estimation:**
**for***t* = 1 to *t_iter_*_2_**do**
**for***i* = 1 to *N*_2_**do**
**◯***_i_*(*t*) = **◯***_i_*(*t* − 1) + *γ***_x_** Σ_*jεS_i_*_ (*δ_ij_* − *d̂_ij_*)**e***_ij_*
**end for**
**end for**
**Path Loss Estimation:**
**for***t* = 1 to *t_iter_*_2_**do**
**for***i* = 1 to *N*_2_**do**
**for***j* = 1 to *N_S_i__***do**
${\hat{\alpha }}_{\mathrm{ij}}(t)={\hat{\alpha }}_{\mathrm{ij}}(t-1)-\gamma_{\alpha }\;\text{log}(10)\frac{P_{0}-{\mathrm{RSS}}_{\mathrm{ij}}}{5}\frac{1}{\alpha_{\mathrm{ij}}^{2}}\delta_{\mathrm{ij}}(\delta_{\mathrm{ij}}-{\hat{d}}_{\mathrm{ij}})$
$\delta_{\mathrm{ij}}=10^{\frac{P_{0}-p_{\mathrm{ij}}}{10{\hat{\alpha }}_{\mathrm{ij}}}}$
**end for**
**end for**
**end for**
**end for**

It is important to note that our proposal requires a higher computational complexity than that required by a pure RSS-based algorithm (without node selection and path loss estimation). In order to reflect this point, we have evaluated the required complexity in terms of number of operations for both cases. In the conventional RSS-based algorithm the total number of operations is equal to:

(13) $$t_{{\mathrm{iter}}_{1}}\times t_{{\mathrm{iter}}_{2}}\times N_{2}\times (2N_{S_{i}}+2)$$

In our proposal, the number of operation is:

(14) $$t_{{\mathrm{iter}}_{1}}\times t_{{\mathrm{iter}}_{2}}\times N_{2}\times ((2N_{S_{i}}+2)+7N_{S_{i}})$$

where the additional term with respect to the former expression is due to the estimation of the path loss. Although it may seem that our proposal results in a larger computational complexity, this is not necessarily the case because our selection mechanism allows us to reduce the number of nodes *S_i_* in each group, possibly leading to a complexity equal to or smaller than that of the conventional RSS-based algorithm. Indeed, for the scenarios addressed here, simulation results reflect that the average number of operations is 18% lower in our proposal than that carried out by the conventional RSS-based algorithm.

## 4. Energy Consumption

Wireless sensor networks nodes rely on low data rates, very long battery life (several months or even years) and very low computational complexity associated with the processing and communication of the collected information across the WSN. In order to maintain the battery life, the reduction of the energy consumption is an important point in WSN.

**Figure 4.**
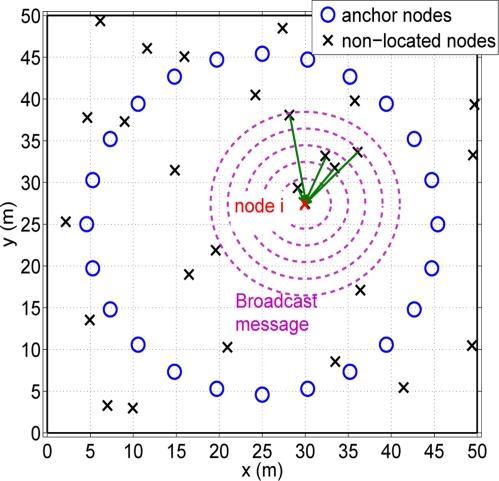
Example of the creation of a group *S_i_*.

Taking into account the localization algorithm presented in the previous section, we can present an energy consumption model based on the required number of transmissions (see Figure 4).

At a first moment each node *i* has to create its own group of cooperating nodes *S_i_*. At the beginning, each node *i* sends a broadcast message with its coordinates **x_i_**. Only those nodes that receive this message answer with their node ID and their location coordinates. With the received messages, each non-located node can create its own *S_i_* group. Once these groups are created, the exchange of messages are only done between cooperating nodes. At this time, the total amount of energy consumed by the network follows the model presented in [17]:

(15) $$ɛ=(\mu_{R_{x}}+\mu_{T_{x}})\left(\sum_{i=1}^{N_{2}}N_{S_{i}}-N_{2}\right)\kappa$$

where *κ* is the number of iterations of the algorithm, *N_S_i__* the number of nodes inside *S_i_*, and *μ_T_x__* and *μ_R_x__* are the energy consumption dedicated for peer to peer transmission and reception procedures, respectively. We have to mention that our model presents some differences compared with the model presented in [17]. We suppose that the energy per transmission is always the same instead of having an energy consumption depending on time. Furthermore, we only take into account the energy consumption at the transmission and reception time. Our node selection mechanism produces a reduction in the number of transmitted messages. For that reason we do not take care about the energy consumption at sensing time, as in the cited reference. Hence, we only present an energy model based on the consumption at the transmission and reception time.

It is worth noting that energy consumption is an increasing function on the number of cooperating nodes (*N_S_i__*). In the next section we propose different node selection methods that allows us to reduce the energy consumption (reducing the number of nodes inside *S_i_*).

## 5. Node Selection Mechanisms

The authors presented in [18] a Node-Selection Least Squares (NS-LS) location algorithm. The idea is to obtain a good trade-off in terms of position accuracy versus energy consumption. As discussed in [18], the derivation of the optimal selection criterion is not possible. For that reason, the authors presented a sub-optimal scheme based on the received power threshold (*RSS_th_*). In other words, only nodes with RSS higher than the *RSS_th_* were allowed for cooperation. This criterion becomes in a simple scheme suitable for a hardware restricted WSN. In particular, the choice of the *RSS_th_* value was designed to assure a minimum number of anchor nodes inside the cooperating nodes group (*S_i_*). In accordance to this value, *N_m_*, different trade-off points in the energy consumption versus accuracy can be achieved. Results showed that *N_m_* = 3 allows the algorithm to achieve an excellent trade-off. Concerning the relation between *RSS_th_* and *N_m_*, we derived in [18] an analytical procedure to obtain the required *RSS_th_* that assures the desired value of *N_m_* To do so, we first considered a uniform distribution for the positions of the non-located nodes and obtained the mean number of anchor nodes inside a circumference of radius *r_th_* as:

(16) $$N_{m}\approx \sum_{j=1}^{N_{1}}\pi r_{\mathrm{th}}^{2}\frac{1}{A}=\frac{N_{1}\pi r_{\mathrm{th}}^{2}}{A}$$

where A is the total area of the considered scenario and *N*_1_ is the total number of anchor nodes in the scenario. Then, by taking into account the existing relation between received power and coverage range radius based on the path loss propagation model, the idea was to select the appropriate received power threshold *RSS_th_* assuring that *N_m_* anchors nodes is inside the coverage range radius. By considering *r_th_* in the expression above as the coverage range radius, we finally established the following relation between *RSS_th_* and *N_m_* (for more in depth explanation see [18]):

(17) $${\mathrm{RSS}}_{\mathrm{th}}={\left(\frac{N_{1}\pi P_{0}^{2/\alpha }}{N_{m}A}\right)}^{\alpha /2}$$

### 5.1. Selection Mechanisms

In this subsection, we derive two node selection algorithm for the proposed cooperative localization scheme. Since in this work we consider the adoption of on-line path loss estimation, we need to adapt the scheme presented in [18] to the new context. The use of RSS thresholds, in particular, is not the optimal choice in an environment where path loss exponents of the different links can be quite different: the node with the highest RSS is not necessary the best one.

**Figure 5.**
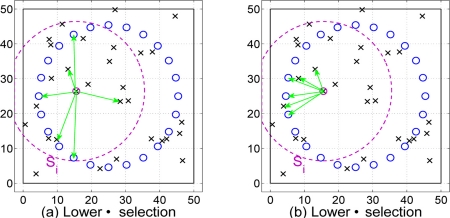
Example of both node selection methods.

In particular we present two node selection mechanisms that depend on the path loss estimates obtained through the Algorithm 1. With those selection mechanisms we try to reduce the number of nodes that cooperates in the location algorithm presented in Section 3 (see Figure 5). The different criteria are: low path loss selection and low distance selection. Hence, both node selection schemes depend on path loss estimates. First, the low path loss selection tries to find the nodes with best channel conditions. Second, the low distance criterion selects those nodes that have a low distance estimate *δ_ij_*. It is possible that these selection criterion could not select a node nearby, because distance estimates could not reflect the reality (*δ* values depend on the quality of *α̂* estimates and the shadowing effects).

- **Low Path Loss Selection** Given all the estimates of *α̂_ij_*, the first selection mechanism selects those nodes that have the lowest values for the path loss exponent. In other words, by sorting the path loss exponents of the nodes inside the coverage of node *i*:
  $${\hat{\alpha }}_{i1}\leq {\hat{\alpha }}_{i2}\leq \ldots \leq {\hat{\alpha }}_{in}1,\ldots ,n\in S_{i}$$
  where *α̂_i_*_1_ and *α̂_in_* are the lowest and highest exponent, respectively. We select the nodes with the lowest values:
  $$S_{i}^{\mathrm{NS}}=\{i1,i2,\ldots ,in_{\alpha }\}$$
  with *n_α_* standing for the number of selected nodes. With this selection mechanism we are interested in the selection of nodes that have better propagation conditions.
- **Low Distance Selection** Now, the idea is to select the closest nodes to node *i*. We want to use nearer nodes in order to reduce distance error estimates, because, as shown in [13], the shadowing effect introduces errors multiplicative to the distance. By sorting now the distance estimate of the nodes inside the coverage of node *i*:
  $$\delta_{i1}\leq \delta_{i2}\leq \ldots \leq \delta_{in}\;1,\ldots ,n\in S_{i}$$
  where in this case *δ_i_*_1_ and *δ_in_* are the lowest and highest distance estimate, respectively. The new group of cooperating nodes becomes:
  $$S_{i}^{\mathrm{NS}}=\{i1,i2,\ldots ,in_{\delta }\}$$
  with *n_δ_* standing for the number of selected nodes.

### 5.2. Selection Mechanisms Performance

**Figure 6.**
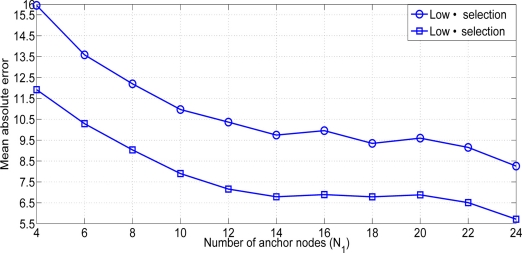
Mean absolute error versus number of anchor nodes (*N*_1_) (*n_δ_* = *n_α_* = 6).

Once the node selection mechanisms are presented, we show the performance of both mechanisms in order to choose the appropriate one. First of all it is necessary to compare both methods. It is shown in Figure 6 that the low distance selection outperforms the low path loss selection in all the scenarios. As we said previously, the selection of those links with a lower value of *α_ij_* could not correspond to the closest nodes. As we are using an RSS-based algorithm, the error introduced at the measurements done at the first phase is multiplicative to the estimated distance. The probability of selecting distant nodes is higher. As a consequence, the performance is affected.

**Figure 7.**
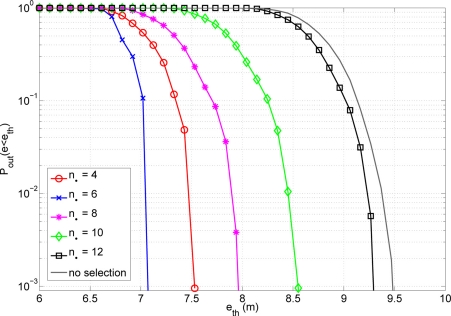
Outage probability (*N*_1_ = 18).

On the other hand, we also have to know which is the optimum number of cooperating nodes. Analytically, it is not straightforward to obtain the value of *n_δ_* that optimize the system behaviour. Then, numerical evaluation is needed to obtain this value. In principle, the optimum number of *n_δ_* is scenario dependent. However, our results have shown that a value equal to 6 is generally the best choice. This means that actually the optimum *n_δ_* does not depend on the fine-grained distribution of the nodes, but rather on general parameters of the scenario (e.g., positioning in 2D or 3D, overall distribution of the nodes in the area under study, *etc.*). It is shown in Figure 7 the probability of outage for different values of *n_δ_*. This probability of outage is the probability of having an error higher than an error threshold. Analysing the results presented, one can see that having a low value of cooperating nodes reduces the possibility of having anchor nodes (anchors are nodes with true information of their exact location) inside the cooperating group. On the other hand, having more cooperating nodes implies the existence of further nodes. This fact implies a higher error of the distance estimates. It is important to achieve a good trade-off between having a group big enough to lodge an anchor but low enough to not use further nodes with a high error at distance estimates. A number of *n_δ_* equal to 6 offers the best results in terms of position accuracy. For that reason we adopt this value in the sequel.

## 6. Numerical Results

This section presents the performance of the proposed location algorithm with on-line path loss estimation and node selection. We present both simulations and experimental results obtained in an indoor scenario. We consider that the path loss exponents take values between a maximum value of 5 and a minimum value of 2 (The uniform distribution of the path loss exponents between 2 and 5 are based on experimental results obtained in [16].). Hence, we simulate path loss values with a uniform distribution (*α ɛ* 𝒰(2,5)). We assume in our algorithm an initial value of the path loss equal to 3.5, which is the middle value of the random values used in the uniform distribution. The experimental parameters are shown in Table 1.

### 6.1. Computer Simulation

**Figure 8.**
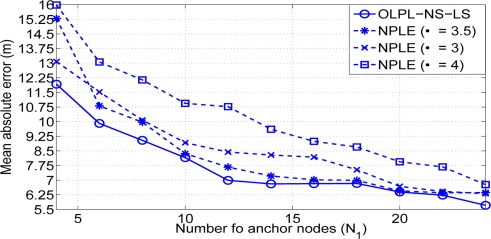
Mean absolute error versus number of anchor nodes (*N*_1_) (*solid line: lower* *distance selection, dashed line: non-path loss estimation*).

The assumption of different path loss exponent for each link allows us to simulate a more realistic scenario to that presented in [18]. With this assumption the importance of doing an on-line estimation of the path loss exponent (*i.e.*, a good propagation model) is reflected in Figure 8. With the proposed solution we achieve a gain in terms of position accuracy that oscillates between 2 and 0.5 meters, compared with that achieved with the Non Path Loss Estimation (NPLE) algorithm. Different values of path loss exponents have been simulated and the best result is always achieved with our on-line path loss estimation and node selection least squares algorithm (OLPL-NS-LS). Having an on-line estimation of the path loss not only has good consequences in the localization performance accuracy but also makes it possible for the algorithm to adapt to possible changes in the scenario. Furthermore, the lowest gain is obtained when the number of anchor nodes is higher than 16 and this is not a usual value in a realistic scenario.

### 6.2. Comparison with Existing Methods

In this subsection we will compare our proposed algorithm OLPL-NS-LS with two different existing solutions: a distributed method based on a Maximum Likelihood algorithm (ML) and centralised algorithm based on Multidimensional Scaling (MDS). We apply the on-line path loss estimation to all the methods in order to achieve a fair comparison between them. Only our OLPL-NS-LS method present the node selection method proposed. We will compare the performance of our method in terms of both energy consumption and positioning accuracy.

The ML localization algorithm [19] used is based on the minimization of the following cost function carried out with a distributed iterative method:

(18) $$C_{\mathrm{ML}}(\text{x})=\sum_{i=1}^{N_{2}}\sum_{jɛS_{i}}{\left({\text{log}}_{10}(\delta_{\mathrm{ij}}(\alpha_{\mathrm{ij}}))-{\text{log}}_{10}(d_{\mathrm{ij}}({\text{x}}_{\text{i}},{\text{x}}_{\text{j}}))\right)}^{2}$$

The MDS algorithm is a simple centralized approach that builds a global map using classical MDS [20]. MDS works well on networks with relatively uniform node density but less well on more irregular networks.

**Figure 9.**
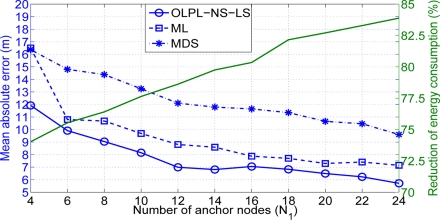
Mean absolute error versus number of anchor nodes (*N*_1_) (*solid line: On-Line Path Loss and Node Selection Least Squares, dashed line: Maximum Likelihood, dash-dotted line: Multi Dimensional Scaling*).

As observed in Figure 9 our OLPL-NS-LS algorithm outperforms the ML localization algorithm. It is important to remark that a gain between 0.5 and 1.5 meters is obtained with our algorithm. This gain is achieved thanks to the selection algorithm. The gain achieved with respect to the MDS localization algorithm is more remarkable. In that case we are comparing a distributed method (our OLPL-NS-LS) with a centralized method (MDS). On the one hand, a centralized method includes more distant nodes. Then, nodes with a high error on their distance estimates are used. On the other hand, all possible nodes inside each group *S_i_* are also used in the path loss estimation process. Probably, these nodes that are not near to a node *i* would not have a similar propagation conditions compared to those nodes that are closer. For that reason a node selection scheme allows to reduce the mean absolute error results in an RSS-based localization algorithm. It is also important to remark on the reduction of the energy consumption. According to our energy consumption model, the use of a reduced cooperating group *S_i_* produces a reduction in the energy consumed by the network. With the use of a number of cooperating nodes *n_δ_* = 6, our OLPL-NS-LS algorithm achieves a percentage of reduction between 74% and 83% compared to the energy consumed by a method without node selection mechanism (see Figure 9).

### 6.3. Experimental Results

**Figure 10.**
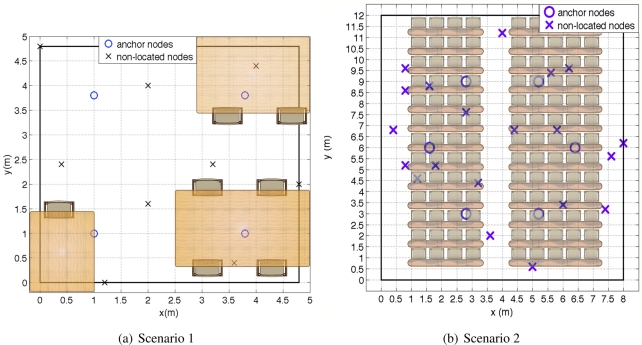
Experimental scenarios.

In order to check the performance in a real scenario, we have carried out measurements in different indoor scenarios with the Mica2 motes @915MHz of Crossbow [21]. We present two different indoor scenarios shown in Figure 10.

At the first scenario the total number of nodes with unknown position, *N*_2_, is 9 (see Figure 10(a)), the number of anchor nodes, *N*_1_, is 4. These nodes are located in a 4.8 m × 4.8 m scenario. The second scenario (see Figure 10(b)) is composed by *N*_2_ = 20, *N*_1_ = 6 and *N*_1_ = 4 in a scenario of 8 m × 12 m.

**Figure 11.**
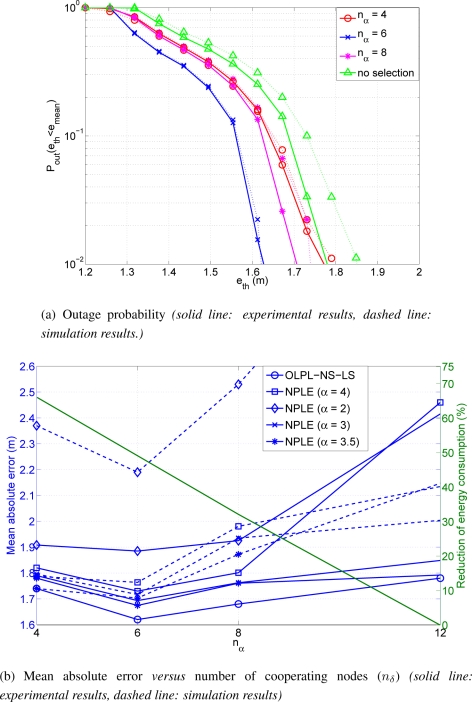
Results at Scenario 1.

**Scenario 1** It is shown in Figure 11(a) the probability of having an error above an error threshold. We present this graphic in order to verify the better results achieved in Figure 7 with only 6 nodes cooperating. We also present simulation results with the same conditions as the real scenario. On the one hand, the best result achieved is with a value of *n_δ_* equal to 6 as achieved in the large scale case. On the other hand, simulation results are very similar to that achieved with the experimental scenario. Furthermore, Figure 11(b) shows the mean absolute error and the percentage of reduction compared to the consumption of the location algorithm without node selection. The best result is again achieved with *n_δ_* equal to 6. With this value, the percentage of reduction in terms of energy consumption is about 50%. As observed, experimental results are quite similar to simulation results in this case. It is also presented the comparison of behaviour between our OLPL-NS-LS and the NPLE with different values of the path loss exponent (*α*). Our OLPL-NS-LS achieves always the best result compare to that achieve with all the NPLE presented (see Figure 11(b)). Best behaviour is achieved with a fixed value of *α* = 3.5. The differences in terms of mean absolute error oscillates between 10 cm when *α* = 3.5 and 0.6 cm when *α* = 2. Results of this experimental scenario show the gains obtained by considering the proposed real time path loss estimation with respect to the case adopting an equal path loss exponent for all the links.

**Figure 12.**
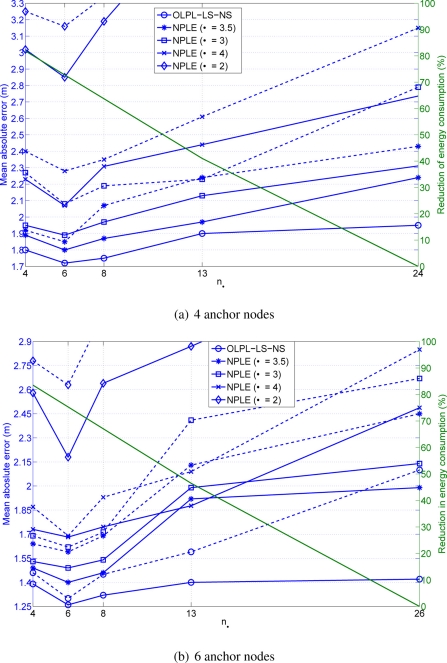
Mean absolute error *versus* number of cooperating nodes (*n_δ_*) *(solid line: experimental results, dashed line: simulation results.)*

**Scenario 2** As commented before, we present two different results: one for a number of 4 anchor nodes and the other for 6 anchor nodes. Figure 12 shows both results. As before experimental and simulation results are presented, showing a similar behaviour between experimental an simulation performance.

It is also remarkable that, as in the previous Figures, the best performance is achieved when the number of cooperating nodes is equal to 6.

We could observe in Figure 12(a) that although we have increased the number of nodes and the scenario dimensions, the performance of the algorithm is similar to that achieved in the scenario shown in Figure 10(a). We have a higher scenario but also more nodes that can be closer. The problem is that the accuracy achieved is equal to 1.7 m in average.

If we observe the results achieved in Figure 12(b), the accuracy obtained is now 1.2 m. Increasing the number of anchor nodes in 2 contributes in a benefit of 0.5 m in the accuracy. Another important point is the benefit of 0.5 m, in terms of accuracy, when we are using our proposal with on-line path loss estimation compared to an algorithm without path loss estimation results. Finally, the reduction in terms of energy consumption is of, approximately, 75% in both scenarios.

In both Figures, one can observe the benefits of the proposed OLPL-NL-LS approach when compared with the case of assuming a constant path loss exponent. We achieve always a better result in terms of position accuracy with our proposal. For example, in the scenario 1 we achieved a minimum difference of 10 cm when *α* = 3.5 and a maximum difference of 1 m when *α* = 2. In the scenario 2 the results show that, when *α* = 3.5 the difference is 15 cm and when the *α* = 2 the difference is 0.9 m. We always achieve a benefit when the path loss is estimated on-line.

## 7. Conclusions

In this paper we have proposed a distributed cooperative RSS-based location algorithm with on-line path loss exponent estimation and node selection. Although RSS-based algorithm constitutes the simplest method, the necessity of having an accurate propagation model is revealed by the results shown. The introduction of a distributed on-line path loss estimation allows the algorithm to infer a good propagation model in a simple way. The need of an off-line calibration is avoided. Furthermore, we have presented an adaptive solution to track possible changes in the environment. Since wireless sensor networks are energy-constrained networks, we have also presented a node selection criterion that reduces the number of cooperating nodes. It has been shown that having a reduced number of cooperating nodes allows us to reduce the energy consumption without affecting the accuracy. Besides, we have presented experimental results that validate the proposed method, and we have compared it with ML and MDS, showing the efficiency in terms of the trade-off between energy consumption and accuracy.

## Figures and Tables

**Table 1.** Simulation Parameters.

**Simulation Parameters**	**Parameter Value**
Size of Sensor Field	50 × 50 m
Number of Non-Located Nodes (*N*_2_)	30
Path Loss Exponent *α_ij_*	2–5
Standard Deviation *σ_v_*	1 dB
First-Meter RSS *P*_0_	−50 dBm
Anchor Radius	20.4 m
Energy Consumption to Transmit or Receive *μ_T_x__* or *μ_R_x__*	400 nJ
